# Autonomic Modulation of Atrial Fibrillation

**DOI:** 10.1016/j.jacbts.2023.03.019

**Published:** 2023-06-28

**Authors:** Wei–Chung Tsai, Tien-Chi Hung, Takashi Kusayama, Seongwook Han, Michael C. Fishbein, Lan S. Chen, Peng-Sheng Chen

**Affiliations:** a Division of Cardiology, Department of Internal Medicine, Kaohsiung Medical University Hospital, Kaohsiung Medical University, Kaohsiung, Taiwan; b Department of Cardiovascular Medicine, Kanazawa University Graduate School of Medical Sciences Kanazawa, Kanazawa, Japan; c Department of Cardiology, Keimyung University Dongsan Medical Center, Daegu, Korea; d Department of Pathology and Laboratory Medicine, David Geffen School of Medicine at University of California-Los Angeles, Los Angeles, California, USA; e Department of Cardiology, Smidt Heart Institute, Cedars-Sinai Medical Center, Los Angeles, California, USA

**Keywords:** neuECG, neuroremodeling, skin sympathetic nerve activity, stellate ganglion, sympathetic nerve activity

## Abstract

The autonomic nervous system plays a vital role in cardiac arrhythmias, including atrial fibrillation (AF). Therefore, reducing the sympathetic tone via neuromodulation methods may be helpful in AF control. Myocardial ischemia is associated with increased sympathetic tone and incidence of AF. It is an excellent disease model to understand the neural mechanisms of AF and the effects of neuromodulation. This review summarizes the relationship between autonomic nervous system and AF and reviews methods and mechanisms of neuromodulation. This review proposes that noninvasive or minimally invasive neuromodulation methods will be most useful in the future management of AF.

## Highlights

• Large SNA often precedes the onset of AF.
• Neuromodulation reduces SNA and controls AF.
• Widely applicable noninvasive or minimally invasive methods of neuromodulation should be developed to control AF.
• More randomized clinical trials are needed.

The autonomic nervous system (ANS) plays a vital role in initiating and maintaining cardiac arrhythmia, including atrial fibrillation (AF).^1^^,^^2^ Simultaneous activation of stellate ganglion (SG) and vagal nerves are associated with the initiation of AF in a canine model.^3^ There is colocalization of sympathetic and parasympathetic nerves in the atrial ganglionated plexi (GP).^4^ Intrinsic cardiac nerve activity, either alone or in collaboration with the extrinsic cardiac nerve activity, is an invariable trigger of paroxysmal atrial tachyarrhythmia (AT) in the canine models.^5^ Although it is possible to directly record the stellate ganglion nerve activity (SGNA) in humans,^6^ that technology has yet to be applied to study the onset of AF. To partially overcome that problem, we developed a method called “neuECG” that simultaneously and noninvasively records skin sympathetic nerve activity (SKNA) and an electrocardiogram (ECG) in both canine models and in humans.^7^, ^8^, ^9^ The skin is well innervated by sympathetic nerves.^10^ In dogs, the postganglionic sympathetic nerve fibers of neck and thorax originate primarily from the SG.^11^ Our canine studies confirmed that the SKNA can be used to estimate the SGNA.^7^^,^^12^^,^^13^ We then performed a human study by inserting a temporary pacing wire into the epicardial fat pad to record the intrinsic cardiac nerve activity after open heart surgery in 11 patients.^14^ We found that low-amplitude SKNA and intrinsic cardiac nerve activity were present at all times, but the burst discharges were observed much less frequently. Figures 2 and 3 of Shen et al^14^ show SKNA and intrinsic nerve activity bursts might occur simultaneously. Both SKNA and burst intrinsic cardiac nerve activity were associated with the onset of premature atrial contraction and premature ventricular contraction and AF. To further investigate the relationship between SKNA bursts and cardiac arrhythmias (including AF), we^15^ recorded neuECG in 10 patients with AF and 6 patients with ventricular tachycardia (VT) or ventricular fibrillation (VF) episodes. Clustering was defined by an arrhythmic episode followed within 1 minute by spontaneous recurrences of the same arrhythmia.

**Figure 1:**
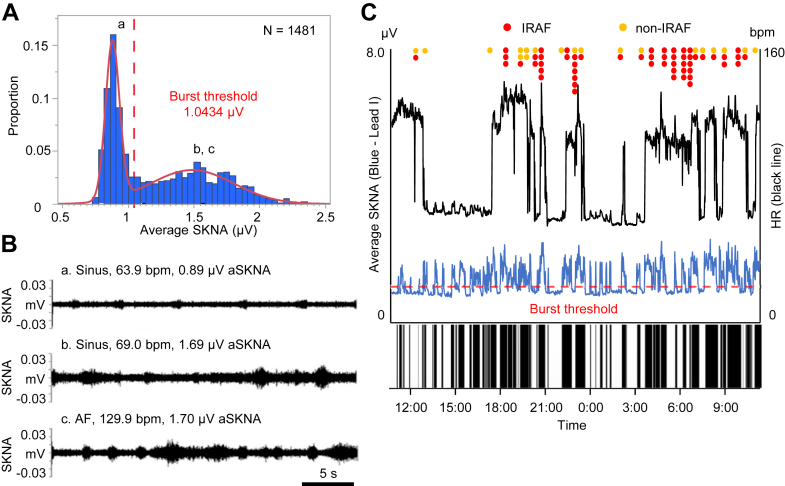
A Method to Objectively Determine the SKNA Bursts (A) The proportion of average skin sympathetic nerve activity (aSKNA) recorded every 60 seconds. The aSKNA indicated 2 Gaussian distributions. The burst threshold is indicated by red dotted line, which was calculated as the mean representing lower amplitude plus 3× SD. (B) Actual recordings of the SKNA in lead I in 30-second window. These recordings correspond to points a (sinus), b (sinus), and c (atrial fibrillation [AF]) in A. Small SKNA discharges occurred regularly in a. Large SKNA bursts were observed irregularly in b and c. (C) Heart rate (black line) and aSKNA from lead I (blue) and plotted over time. The immediate recurrence of atrial fibrillation (IRAF) was defined as a reinitiation (recurrence) of AF within 1 minute after the termination of a prior AF episode. IRAF is indicated by red while other AF episodes are orange (non-IRAF). The large and frequent SKNA bursts occurred during AF. There were smaller bursts of nerve activities associated with sinus rate acceleration. The binary time series graph shows the SKNA burst (black) versus nonbursting period (white), indicating SKNA bursts preceded the AF clustering episodes. From Kusayama et al^16^ with permission. bpm = beats/min.

To better understand the relationship between SKNA bursts and arrhythmia clustering, we^9^^,^^16^ developed a new method to objectively determine the SKNA bursts. Figure 1 illustrates the method using a 24.7-hour long recording. We first analyzed the average voltage of skin sympathetic nerve activity per sample (aSKNA) in each 60-second window. We then plotted the histogram of aSKNA that shows the proportion of windows with a given amplitude (Figure 1A). There are 2 groups of data; each can be fitted with a Gaussian distribution. The first (left) Gaussian distribution represents the baseline nerve discharges, and the second (right) Gaussian distribution represents the burst activity. We used the mean plus 3× SD of the first Gaussian distribution (1.0434 μV) as the threshold to separate these 2 groups of activities. Figure 1B shows actual recordings at points a, b, and c in Figure 1A. Figure 1C shows continuous recording over the same period as shown in Figure 1A. The onsets of AF were indicated by red dots for immediate recurrence of atrial fibrillation (IRAF) and orange dots for non-IRAF. The IRAF was defined as the recurrence of AF within 1 minute from the termination of a previous episode. The horizontal dotted red line indicates the threshold for burst determination (1.0434 μV). The leading edge and trailing edge of each burst was automatically determined. The binary time series graph at the bottom of Figure 1C shows the SKNA burst (black) versus nonbursting period (white). There was a total of 68 SKNA bursts in the entire recording. Using that method, we showed that the total duration of SKNA bursts associated with AF was longer than that associated with sinus rhythm, indicating elevated sympathetic tone during AF. We found that there was a latency between the onset of SKNA bursts and the onset of AF, with a median of 9.0 (IQR: 5.0-15.5) minutes. In the same study, we also observed a 3-minute latency between SKNA bursts and the onset of VT. The presence of this latency implies that there needs to be a sufficient accumulation of neurotransmitters locally to induce an episode of AF. To achieve that effect, large and sustained SKNA bursts are needed for AF induction.

## Skna Bursts and the Termination of AF or AT

While studying the association between SKNA bursts and the onset of AF, we unexpectedly discovered that there is also an association between SKNA bursts with the termination of AT. That study^17^ included 11 patients, including 3 with AT and 8 with AF. One of the patients with AT had a total of 32 episodes of AT. We observed that whereas active SKNA bursts are present prior to the onset of AT, there are also large SKNA bursts and transient heart rate acceleration immediately preceding the termination of AT. Because this phenomenon was observed in only 1 of 3 patients with AT studied, its clinical importance remains unclear. Similarly, in all 8 patients with paroxysmal AF, large SKNA bursts were frequently, albeit not always, present before the AF termination. Because SKNA bursts often occurred throughout the period of AF perpetuation, it was difficult to conclude that these SKNA bursts were responsible for termination of AF. A potential clinical implication of these findings was that suppression of the SKNA bursts might prevent AF initiation, but suppression might also prevent AT/AF termination in some patients.

## The Potential Effects of AF on SNA

The rapid ventricular rate and irregular cycle length during AF can lead to intermittently reduced blood pressure, which is known to increase the muscle sympathetic nerve activity (SNA).^18^ In canine models, the SGNA was significantly elevated 15 seconds before and remained elevated 30 seconds after the onset of the atrial arrhythmia.^3^ In humans, large and sustained SNA were associated with the temporal clustering of AF and VT/VF.^16^ These findings suggest that AF itself may increase the SNA, which in turn triggers the next episode of AF and leads to AF cluster. Because the relationship of AF with the SNA may be bidirectional, reducing SNA by neuromodulation may reduce both the frequency and the total burden of AF.

## Cardiac Afferent Innervation and AF

Cardiac afferent reflexes are abnormal in patients with AF during sinus rhythm and dysfunctional during AF.^19^ In canine models, apnea increases GP activity, followed by vagal bursts and tonic SG firing.^20^ Resiniferatoxin decreases SNA and GP nerve activity and abolishes apnea's electrophysiologic response and AF inducibility. These data imply that sensory neurons and afferent nerves play a role in apnea-induced AF. However, because it is difficult to selectively record afferent nerves in ambulatory animals or humans, the direct relationship between afferent nerve firing and onset of AF remains unclear.

## Skna Elevation and MI

**Figure 2:**
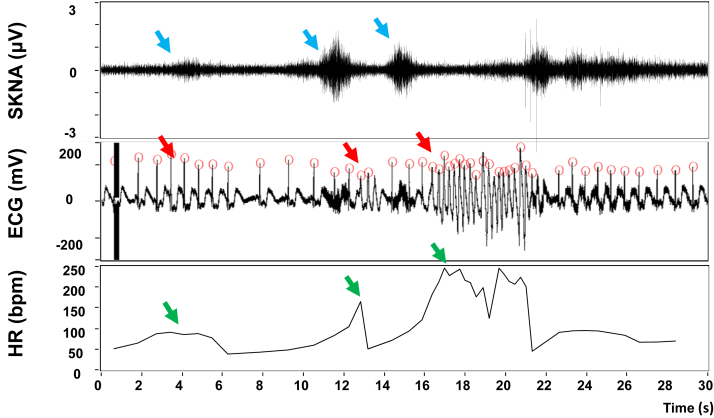
SKNA Bursts and Nonsustained Ventricular Arrhythmia in a Patient With STEMI (Top) The blue arrows indicate the nerve bursts. (Middle) The red arrows indicate the corresponding electrocardiogram (ECG) changes. (Bottom) The green arrows indicate the corresponding heart rate changes. The first nerve activity induced a burst of atrial tachycardia and heart rate acceleration. The second nerve activity induced a nonsustained ventricular tachycardia (3 beats). The third nerve activity induced a longer nonsustained ventricular tachycardia episode (20 beats). From Huang et al^26^ with permission. STEMI = ST-segment elevation myocardial infarction; other abbreviations as in Figure 1.

A vast majority of patients with AF (82%) have either obstructive or nonobstructive coronary artery diseases.^21^ A higher burden of coronary artery disease within all arteries supplying blood flow to the atrial myocardium are associated with higher odds of new-onset AF at 1 year.^22^ In addition to ischemia, myocardial infarction (MI) is followed by cardiac nerve sprouting, sympathetic hyperinnervation, and elevated SNA both in canine models and in humans.^23^, ^24^, ^25^ The elevated sympathetic tone can contribute to the development of AF and other types of cardiac arrhythmias. We recently performed a study to prospectively measure the SKNA in 128 patients with acute coronary syndrome (ACS) and compare the magnitude with that of control subjects.^26^ The neuECG was recorded with ECG lead I configuration at baseline, during mental math stress, and during recovery. Approximately 55% of patients with ACS have ST-segment elevation myocardial infarction (STEMI) and 45% have non–ST-segment elevation ACS. Ventricular arrhythmias were seen in 18% of the patients with ACS. The primary findings of the study, as shown in Table 1, were that the baseline, stress, and recovery average SKNA were higher in ACS than in control subjects. The age of the patients was positively associated with aSKNA. The SKNA in the ACS subject showed more nerve bursts in each phase than in the control subjects. Among patients with ventricular arrhythmias, 15 (83%) had nonsustained VT, 1 (6%) had sustained VT, and 2 (11%) had VF. Figure 2 shows a recording from a patient with STEMI. Large SKNA bursts correlated with the occurrences of VTs. After adjusting for the potential confounders, the ventricular arrhythmias occurrence elevated by 23% when aSKNA level elevates by 0.1 μV and by 8.33-fold if aSKNA level elevated by 1 μV.

**Table 1:** The Average SKNA in Control Group and ACS Group

	Control	ACS	Difference	*P* Value
	Mean ± SD	Mean ± SD	Adj. β ±SE a	
Baseline aSKNA, μV	0.77 ± 0.22	1.05 ± 0.35	0.26 ± 0.04	<0.001^b^
Stress aSKNA, μV	1.18 ± 0.36	1.34 ± 0.37	0.15 ± 0.06	0.012^b^
Recovery aSKNA, μV	0.82 ± 0.27	1.13 ± 0.31	0.30 ± 0.05	<0.001^b^
Stress/baseline aSKNA ratio	1.57 ± 0.41	1.35 ± 0.30	-0.20 ± 0.06	0.002^b^
Stress/recovery aSKNA ratio	1.49 ± 0.42	1.21 ± 0.25	-0.27 ± 0.06	<0.001^b^
Recovery/baseline aSKNA ratio	1.08 ± 0.20	1.15 ± 0.22	0.07 ± 0.04	0.069

ACS = acute coronary syndrome; adj. β = adjusted mean; aSKNA = average skin sympathetic nerve activity.

a Adjusted β were obtained from multiple linear regression models adjusted for age, sex, cigarette smoking, alcohol drinking, and betel-quid chewing.

b *P* < 0.05 for significant difference between ACS and control. From Huang et al^26^ with permission.

There were sex differences of SKNA among control subjects but not among patients with ACS. In control subjects, aSKNA was higher in women than in men at baseline and recovery phases but not during mental stress. These findings suggest that sympathetic reserve (stress/baseline) is higher in men than in women. In comparison, the aSKNA was elevated in both men and women during ACS but there were no sex differences at baseline, during stress, and after recovery. Another finding of the study is that the aSKNA was correlated with systemic norepinephrine concentration. Blood samples collected from ACS (n = 20) and control (n = 20) groups were used to study the association between aSKNA (μV) and systemic plasma norepinephrine level (ng/mL). After adjusting for age, the aSKNA was positively correlated with plasma norepinephrine level (*R*^2^ = 0.20, β = 0.65 ± 0.23 μV per ng/mL*; P =* 0.007). Heightened sympathetic nerve activity likely contributed to the elevated norepinephrine levels.

## Effects of Neuromodulation

### Renal denervation

Renal denervation (RDN) is an extensively studied neuromodulation procedure both for hypertension and for AF control. Relevant to this review, Steinberg et al^27^ performed a multicenter, single-blind, randomized clinical trial that included 302 randomized participants. The investigators found that among patients with paroxysmal AF and hypertension, RDN added to catheter ablation, compared with catheter ablation alone, and significantly increased the likelihood of freedom from AF at 12 months. The HR was 0.57, indicating a highly clinically significant effect. A meta-analysis comparing pulmonary vein isolation (PVI) alone with PVI + RDN in patients with hypertension also suggested a significant beneficial effect of RDN in controlling AF.^28^ The role of RDN in AF is currently strongest in those with concomitant hypertension. These data do not rule out the possibility that RDN achieved AF control by modifying hypertension, a known risk factor for AF.^29^

### Effects of RDN in normal dogs

**Figure 3:**
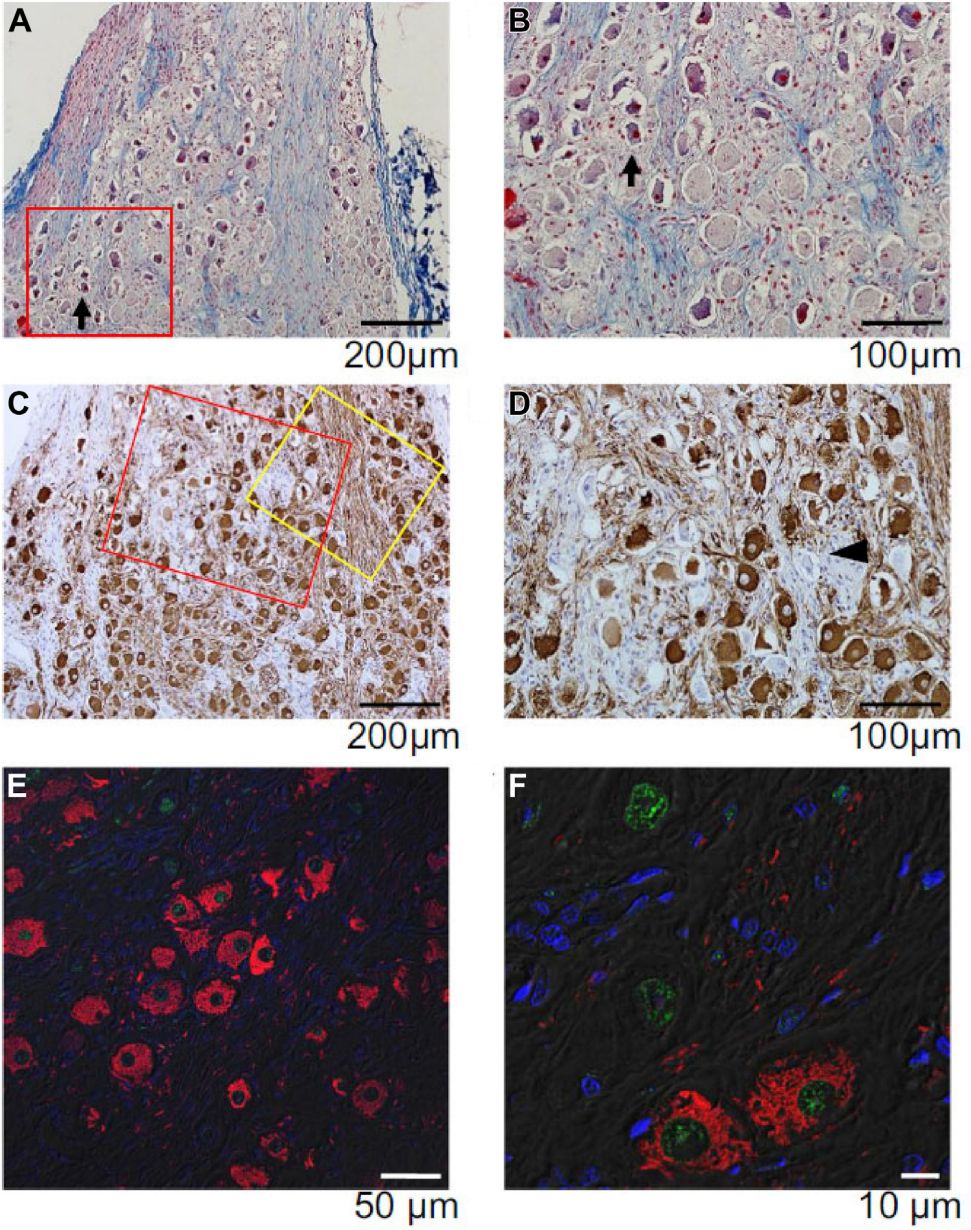
Histology of the Left SG in a Dog With Bilateral RDN (A,B) Masson trichrome staining of the SG. The black arrow indicates injured ganglion cells with pyknotic nuclei and contracted cytoplasm (B). (C,D) Tyrosine hydroxylase (TH) staining of the SG. The black arrowhead points to TH-negative ganglion cells (D). (E,F) The stellate ganglion (SG) with terminal deoxynucleotidyl transferase dUTP nick end labeling (TUNEL) and TH double staining from the yellow box area (C) by confocal microscopy. TUNEL (green) positive ganglion cells were observed. Some TUNEL-positive ganglion cells had pyknotic cytoplasm and stained negative for TH (F). From Tsai et al^30^ with permission. RDN = renal denervation.

**Figure 4:**
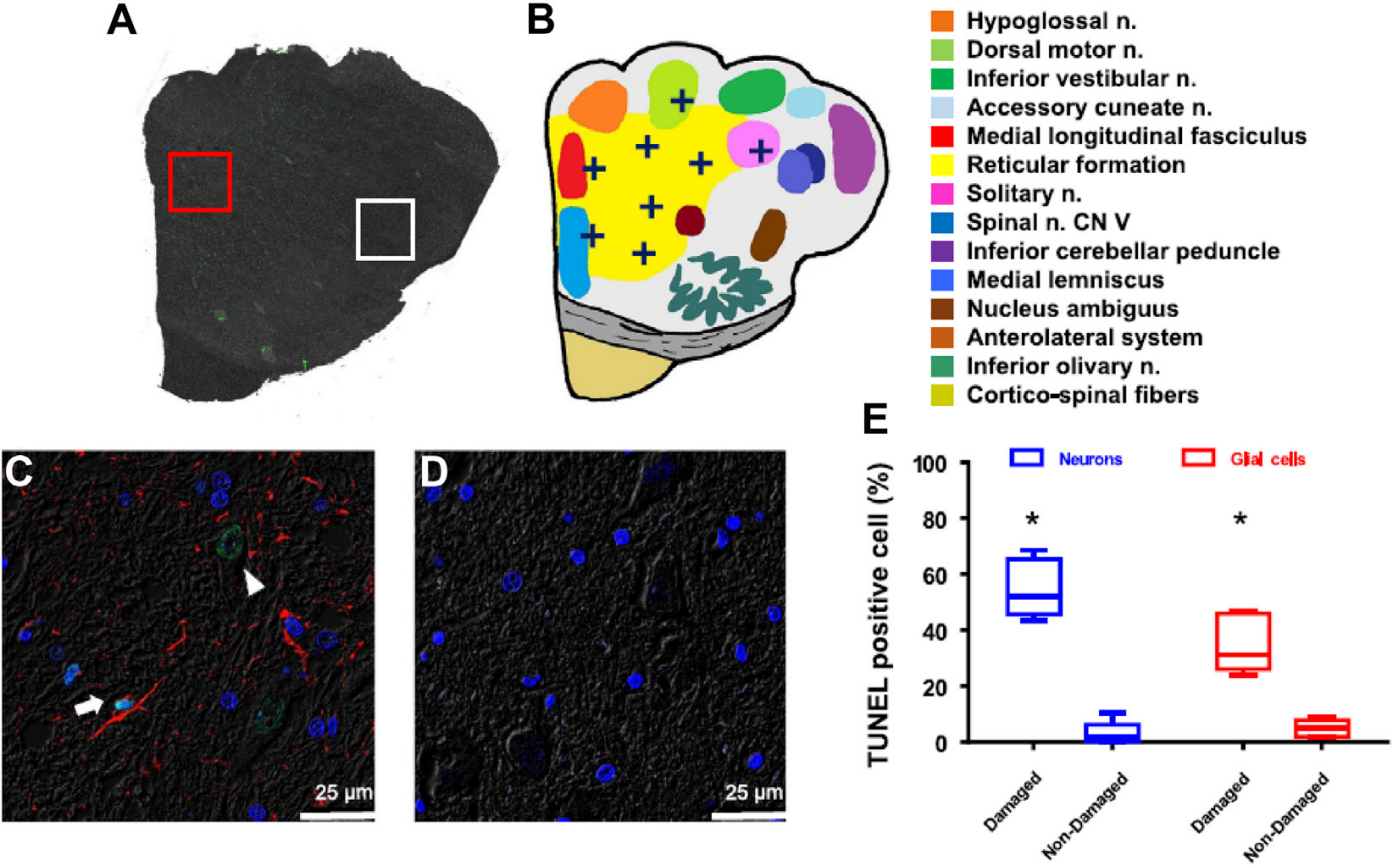
Immunofluorescence Microscopy Images of the Brainstem at Level 1 in a Bilateral RD Dog (A) Confocal microscope image of terminal deoxynucleotidyl transferase dUTP nick end labeling (TUNEL) staining of the entire left half of the brainstem by combining images taken with 10× objective. The TUNEL positivity (green) was mostly distributed in the medial half of the brainstem. (B) Schematic of TUNEL positivity (dark blue cross) in different color-coded structures. (C) TUNEL and glial fibrillary acidic protein (GFAP) double staining in high TUNEL-positivity area of A (red box). Green indicates positive TUNEL stain, red indicates positive GFAP stain, and blue is the 4′,6-diamidino-2-phenylindole stain of the nuclei. An arrowhead points to a TUNEL-positive neuron and an arrow points to a TUNEL-positive glial cell. There was high level of glial reaction as indicated by the strongly positive GFAP staining. (D) The same staining of the white box area in A. There were no TUNEL-positive or GFAP-positive cells in that region. (E) The percentage of TUNEL-positive neurons in “damaged area” and “nondamaged area” in bilateral renal denervation dogs. The percentage of TUNEL-positive neuron cells significantly increased in “damaged area.” ([A] Scanning and merging of 100× images; [C,D] 800×). From Tsai et al^30^ with permission. CN V = trigeminal nerve.

We hypothesized that the mechanisms of RDN might result from the remote neuromodulation that involves both the left SG and the brainstem.^30^ In that study, 9 normal dogs underwent a radio transmitter implantation to record SGNA and were monitored for 2 weeks before and 2 months after radiofrequency RDN procedure. We found that bilateral RDN caused significant central and peripheral sympathetic nerve remodeling and reduced SGNA. The SG and the brainstem remodeling were documented by histologic examinations. Figure 3 shows the histologic changes in the SG after bilateral RDN. Figures 3A and 3B show increased fibrosis and cell death (Masson trichrome stain). Figures 3C and 3D show a significantly increased number of tyrosine hydroxylase–negative neurons in the SG. Figures 3E and 3F show positive terminal deoxynucleotidyl transferase dUTP nick end labeling (TUNEL) staining, documenting cell death. Figure 4 shows the histologic changes in the brainstem, with neurons stained positive for TUNEL. In addition, we performed ^18^F-2-fluoro-2-deoxyglucose uptake study in 2 dogs. The results show that there were −16% and −13% changes in ^18^F-2-fluoro-2-deoxyglucose uptake, respectively, in pons and medulla. These findings indicate that there are both SG and brainstem remodeling after RDN.

### RDN in dogs with acute MI

**Figure 5:**
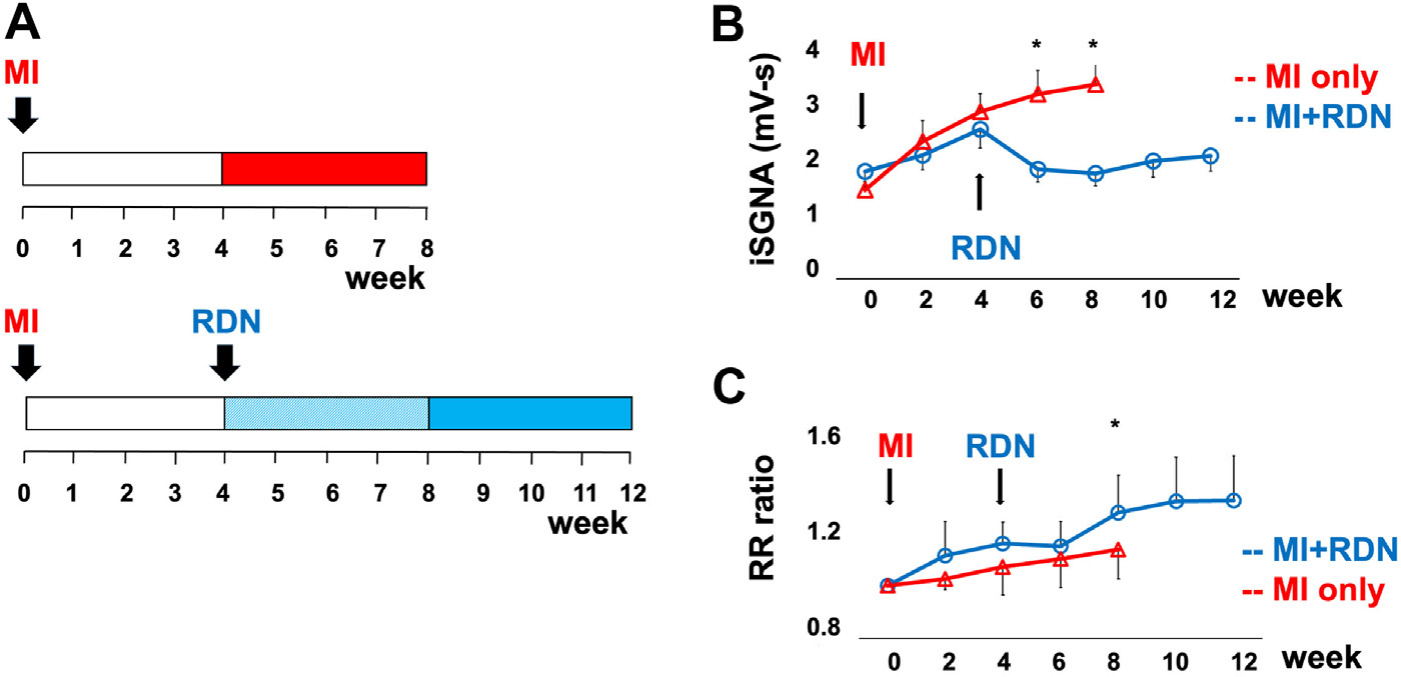
RDN After MI (A) The protocol of renal denervation (RDN) and myocardial infarction (MI) creation in protocols 1 (MI only) and 2 (MI+RDN). In the MI group, we created MI and followed for 8 weeks. In the RDN group, the dogs had MI followed by RDN at week 4 and were followed for an additional 8 weeks. Week 0 is the week immediately after MI. (B) RDN on integrated stellate ganglion nerve activity (iSGNA) over time, showing RDN reduced the iSGNA. (C) The affects of RDN on RR interval ratio compared with week 0. The RDN group had an accelerated RR ratio lengthening (slowing of heart rate) compared to the MI-only group.

**Figure 6:**
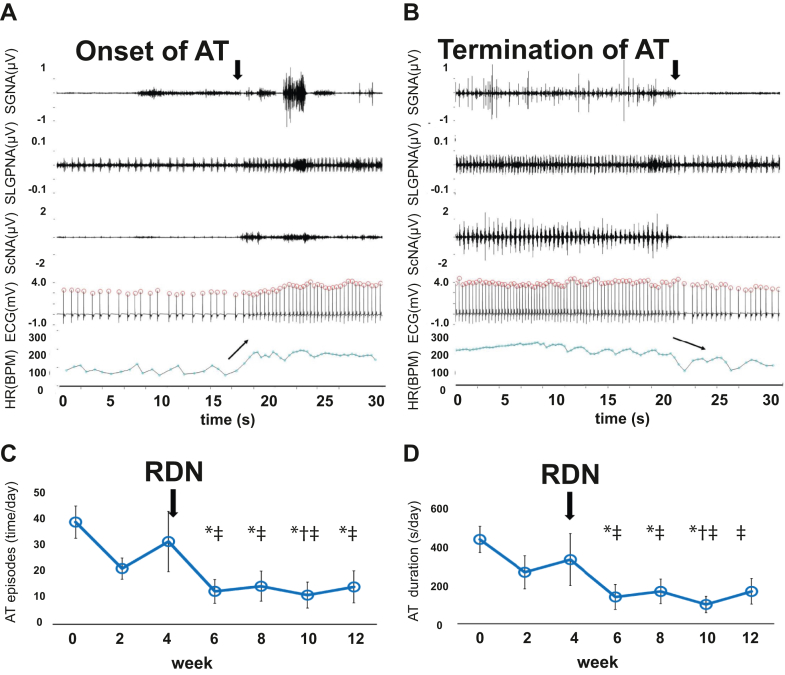
Effects of RDN on ATs (A, B) The onset and termination of atrial tachyarrhythmias (ATs), respectively. A long episode of stellate ganglion nerve activity (SGNA) preceded the onset of AT. Termination of SGNA was coincidental with the termination of AT. The superior left ganglionated plexi nerve activity (SLGPNA) activity was stable before the onset of AT, but then increased only after the onset of AT (perhaps as a reaction to AT) along with a large burst of SGNA. These findings suggest that the arrhythmia itself evokes a substantial change in autonomic activity. With regard to termination, it is possible that cessation of SGNA or SLGPNA led to termination of AT. However, it is also possible that the AT termination resulted in reduction of nerve activity. (C,D) The AT episodes and the AT duration during the study in the MI+RDN group. Note that RDN reduced both the AT episodes and duration. ∗*P* < 0.05 vs week 0; †*P* < 0.05 vs week 2; ‡*P* < 0.05 vs week 4. ScNA = subcutaneous nerve activity; other abbreviations as in Figures 1 to 3, and 5.

Because acute MI significantly increases sympathetic tone and cardiac arrhythmia, it is an excellent model to determine whether RDN is antiarrhythmic. Yamada et al^31^ performed RDN in a rabbit model of MI and heart failure by coronary ligation. They found that RDN reversed atrial electrical and structural remodeling and suppressed AF inducibility. Zhang et al^32^ reported that RDN in a canine model of MI decreased whole-body and local tissue sympathetic activity and reversed neural remodeling in the heart and SG. We carried out a similar study to investigate the effects of RDN on MI in 11 dogs (Figure 5A) (Unpublished data, 2023). The MI was created by left circumflex artery ligation at week 0. All dogs had SGNA, subcutaneous nerve activity, and superior left GP nerve activity monitored by the implanted D70-EEE radio transmitter (Data Sciences International). The control group (n = 5) had MI only and was followed for 8 weeks. The experimental group (n = 6) underwent RDN 4 weeks after MI and was followed for an additional 8 weeks. We used the ratio of integrated SGNA after MI and at baseline to gauge the changes of sympathetic tone. At the end of follow-up, the ratio was 1.78 (95% CI: 1.34-2.22) in the control group, which was significantly (*P =* 0.004) higher than that of the RDN group (0.94; 95% CI: 0.86-1.02) (Figure 5B). The ratio of RR interval at 2 months and RR interval at baseline was significantly (*P =* 0.030) shorter in the control group (1.14; 95% CI: 0.98-1.29) than in the RDN group (1.31; 95% CI: 1.20-1.43) (Figure 5C). These dogs also have a significant number of spontaneous AT episodes, characterized by abrupt onset and termination of the atrial arrhythmias. Figure 6A shows an example of SGNA followed by AT onset (arrow). Figure 6B shows the termination of AT after SGNA activity cessation. Figures 6C and 6D show that RDN decreased the number of AT episodes and duration. Both the present study and the study by Zhang et al^32^ showed that RDN reduced sympathetic tone. These effects led to a reduction of ventricular arrhythmias in the Zhang et al^32^ study and a control of AT episodes in our study.

### Possible mechanisms of neural remodeling after RDN

Dying back neurodegeneration is introduced by either genetic lesions or toxic condition–induced axonal loss.^33^ The “dying back” hypothesis proposes that degeneration of each axon starts at the distal end and moves retrogradely.^34^ Alzheimer disease, Parkinson disease, and amyotrophic lateral sclerosis are among the diseases associated with dying back degeneration.^35^ Injury-induced axon degeneration has been used extensively to study axon degeneration.^35^ Those studies have identified molecular mechanisms that act either to protect or to promote degeneration. We reported that following RDN, an average of 40% of the ganglion cells in the right and left SG are TUNEL-positive, indicating cell death.^30^ We also observed brainstem remodeling in dogs that underwent RDN. These changes are best explained by injury-induced dying back degeneration. The process of transneuronal degeneration then induced brainstem remodeling.^36^ However, we do not have definitive proof that these processes are responsible for all the histologic changes observed in the SG and the brainstem.

### GP and ligament of Marshall ablation

Sympathetic nerves from the middle cervical and SG pass along the ligament of Marshall (LOM) to innervate the left ventricle.^37^ GP modulate the autonomic interactions between the extrinsic and intrinsic cardiac ANS.^38^ Both the GP and the LOM are targets during catheter ablation of AF.^39^ Zhao et al^40^ created an AF model in dogs by intermittent rapid atrial pacing to evaluate the effects of superior left GP and LOM ablation on SG remodeling. The results showed that superior left GP and LOM ablation causes significant left SG remodeling and cell death, with histologic findings similar to those observed after RDN. These findings suggest that retrograde (dying back) axon degeneration may occur after superior left GP and LOM ablation, leading to reduced sympathetic outflow. Because we only ablated the superior left GP and LOM in the latter study, whether SG remodeling occurs with other cardiac GP ablation remains unclear. A randomized clinical trial showed that in addition to PVI, autonomic denervation achieved by anatomic main left atrial GP ablation confers a significantly higher success rate compared to either PVI or GP ablation alone in patients with AF.^41^ However, the GP ablation is technically complex. Only 7% of the experts routinely employ GP ablation during AF ablation procedures.^39^

### Botulinum toxin injection

Botulinum toxin is widely used clinically to treat various diseases.^42^ It irreversibly inhibits release of acetylcholine at the neuromuscular junction and in cholinergic sympathetic and parasympathetic neurons. Because it takes nerve sprouting to recover nerve function, the therapeutic effects may last for 2-3 months. Claus et al^43^ reported that repeated intramuscular botulinum toxin injection causes a significant attenuation of heart rate variability parameters that lasted up to several months. Direct injection of botulinum toxin to the SG can inhibit left SG function and improve cardiac remodeling in a large animal model of chronic MI.^44^ Lee et al.^45^ used botulinum injection into the celiac plexus for celiac plexus block and found it is an effective alternative to RDN in managing refractory hypertension. Whereas botulinum has significant and prolonged functional effects, direct botulinum injection into the superior cervical ganglion in rabbits did not cause significant histologic changes.^46^ Because it is thought that neurotoxin application to the nervous structures can modulate them, randomized clinical trials have been conducted to study the effects of botulinum toxin on postoperative AF. Although some studies show significant beneficial effects during the postoperative period and during long-term follow-up,^47^, ^48^, ^49^ a subsequent larger study did not reproduce these positive results.^50^ A common limitation of these studies is that the mechanisms of postoperative AF is complex.^51^ Because inflammation, ischemia, and other factors may contribute to the onset of postoperative AF, neuromodulation alone may not be effective in preventing its occurrence. The negative results may not be applicable to ordinary AF, which is more dependent on ANS activity as its triggers. However, because botulinum toxin injection does not cause histologic changes, its affects may be short-lasting and may not be ideal for permanent neuromodulation.

### Rapid electrical stimulation

The excessive use of axons may lead to intracellular Ca^2+^ overload, a common mechanism of axonal degeneration and cell death.^52^^,^^53^ That is probably why sustained electrical stimulation of the perforant path causes epileptic brain damage in rats.^54^ Both the dendritic and somal degenerative changes in that study^54^ closely resemble the “excitotoxic” type of damage that the putative transmitters glutamate and aspartate are known to cause. Based on those findings, we hypothesized that rapid electrical stimulation of the peripheral sympathetic axons can modulate/damage the sympathetic neurons in the stellate and other ganglia, leading to reduced sympathetic outflow and better arrhythmia control. Our laboratory has tested this hypothesis using several different models.

#### Cervical vagal nerve stimulation

Vagal nerves contained a significant amount of sympathetic nerve fibers.^55^, ^56^, ^57^ Our canine experiments showed that low-level vagal nerve stimulation (VNS) could suppress SGNA and the incidences of paroxysmal AT in ambulatory canines.^58^ This was followed by another study of VNS during sustained AF in a canine model.^59^ We found that VNS reduced SGNA and ventricular rate. A histologic examination of the left SG showed extensive damage. In the damaged region, the cells showed pyknotic nuclei, reduced tyrosine hydroxylase staining, increased percentage of tyrosine hydroxylase–negative ganglion cells, and positive TUNEL staining. Because VNS is clinically used to control drug-resistant epilepsy, we recorded SKNA in 26 patients with drug-resistant epilepsy who were admitted for video electroencephalographic monitoring. Among them, 6 were treated with VNS and 20 were not. We found that patients with VNS had significantly lower SKNA than those without VNS. These findings are consistent with the effects of VNS in canine models. Because reduced ventricular rate was observed while the stimulator was off, the rate control was not achieved by the direct activation of the parasympathetic nerves.

#### Subcutaneous nerve stimulation

In addition to innervating the heart, the SG also innervates the cutaneous nerves of the neck and the upper thorax.^11^ We hypothesized that stimulating the subcutaneous sympathetic nerves in those areas would have the same effects as stimulating the sympathetic components inside the vagal nerve. To test that hypothesis, we performed a series of studies in canine models by using implanted VNS devices to stimulate the subcutaneous sympathetic nerves. In the first study,^60^ we performed subcutaneous nerve stimulation (ScNS) at the Xinshu acupoint and at the left lateral thoracic nerve for 2 weeks. The results show significant SG remodeling and suppression of paroxysmal AT in ambulatory dogs. We then performed a study to test the hypothesis that ScNS can control the ventricular rate in persistent AF. We found that thoracic ScNS remodels the SG, slows the ventricular rate, and preserves left ventricular systolic function in the persistent AF.^61^ Contrary to that observed in renal denervation, the ^18^F-2-fluoro-2-deoxyglucose uptake in the pons and medulla was significantly higher (rather than lower) in the ScNS group than in the sham control group at the end of the study.^61^ An important factor determining the response to stimulation is the strength of the stimulation. Low strength of stimulation delivered to the SG is proarrhythmic because it can cause nerve sprouting and sympathetic hyperinnervation.^62^ Consistent with the results of that study, we found that low-output (0.25 mA) ScNS is proarrhythmic by increasing cardiac sympathetic nerve sprouting, plasma norepinephrine concentration, and the duration of AT, whereas the high-output (2.5 mA and 3.5 mA) stimulation had the opposite effects.^63^ A limitation of the above-mentioned studies is that we performed an incision to look for subcutaneous nerve structures for stimulation. The need for a several centimeter-long incision may prevent clinical translation. We hypothesize that because skin is well innervated by the sympathetic nerves, a blindly inserted electrode might be able to stimulate the sympathetic nerves and cause SG remodeling. We performed a canine study to test that hypothesis.^64^ We demonstrated that ScNS delivered with blindly inserted electrodes can also improve ventricular rate control and reduce atrial fibrosis in the persistent AF model in dogs. We are currently conducting a prospective randomized clinical trial (STALL-AF [Using Electrical Nerve Stimulation to Control Atrial Fibrillation]; NCT04529941) to determine whether ScNS through a blindly inserted electrode can control AF in humans.

#### Tragus stimulation

Because the cutaneous layer of external auditory canal (Ramsay Hunt zone) was innervated by a sensory branch of the X (10th) cranial (vagus) nerve, transcutaneous electrical stimulation of the nervous system on the Ramsay Hunt zone might also stimulate the vagal nerve and therefore is beneficial for seizure control.^65^ Yu et al^66^ performed low-level tragus stimulation in a canine model and showed that it reverses AF inducibility, suggesting a potential noninvasive treatment of AF. The term “low level” is used to describe a voltage level that does not slow the sinus rate or atrioventricular conduction. In that first canine study, the average threshold was 9.8 V. Assuming the impedance is ∼1,000 Ω, the strength of stimulation is high enough to cause neural remodeling. Stavrakis et al^67^^,^^68^ attempted to translate these findings to humans. They showed that noninvasive tragus stimulation was associated with 85% lower AF burden than the ear lobe stimulation.^69^ However, the positive results were more likely caused by the increased AF burden in the control group than the reduction of AF burden in the experimental group. Another randomized clinical trial showed that Tragus stimulation reduces myocardial ischemia-reperfusion injury in patients with STEMI.^70^ These encouraging preliminary results suggest that Tragus stimulation might be useful in AF control, but more studies are needed to determine the mechanisms of action and to support that conclusion.

## Future Directions

Increased sympathetic nerve activity plays an important role in initiating AF. Neuromodulation methods may reduce sympathetic outflow and achieve AF control. Small randomized clinical trials suggested that neuromodulation methods might be useful in AF control in highly selected patient populations, but the results have not been widely reproduced. Furthermore, none of the studies were powered to demonstrate a mortality benefit. Because AF is a chronic disease that affects a large number of patients, even a small benefit might have a significant impact on public health. Finding a neuromodulation procedure that has a small benefit to a large number of patients might be as important as finding a procedure that delivers a large benefit to a small group of highly selected patients.

We propose that both paroxysmal and persistent AF may benefit from neuromodulation. In patients with paroxysmal AF, the AF burden may be reduced by suppressing triggers and reducing the secondary elevation of the SNAs that help maintain AF. Neuromodulation may also result in better rate control in persistent/permanent AF.

## Conclusions

ANS plays an important role in the initiation and maintenance of cardiac arrhythmias. Neuromodulation procedures can reduce sympathetic outflow through axonal damage and dying back degeneration or through electrical stimulation–induced intracellular calcium accumulation and cell death (See Central Illustration). Transneuronal degeneration may further magnify the effects of neuromodulation procedures by remodeling the remote nerve structures. Although randomized clinical trials have suggested that neuromodulation could improve the outcomes of catheter ablation, the challenge is to find a noninvasive or minimally invasive neuromodulation method that can be easily applied to all patients with AF.

**Central Illustration:**
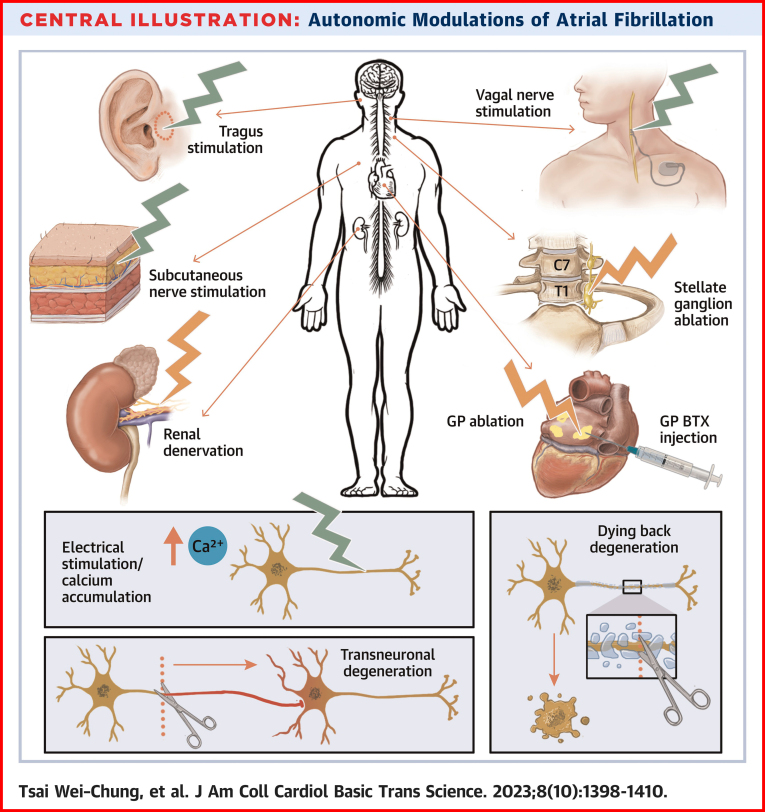
Autonomic Modulations of Atrial Fibrillation

## Funding Support and Author Disclosures

This study was supported in part by the Ministry of Science and Technology, Taiwan (grant MOST 110-2314-B-037-111), the Kaohsiung Medical University Hospital Research Foundation (grants SI11001, SI11101, KMUH105-5M07, KMUH-S10707, and NK111P24), the US National Institutes of Health (grants R01HL139829 and OT2OD028190), and the Burns and Allen Chair in Cardiology Research, Cedars-Sinai Medical Center, Los Angeles, California. Dr Peng-Sheng Chen and Lan S. Chen are co-inventors of U.S. Patents awarded to Indiana University. All other authors have reported that they have no relationships relevant to the contents of this paper to disclose.
